# From burnout to engagement: enhancing the wellbeing and performance of conservationists

**DOI:** 10.3389/fvets.2025.1567931

**Published:** 2025-09-19

**Authors:** Thirza A. C. Loffeld, Simon A. Black, Tatyana Humle

**Affiliations:** ^1^School of Anthropology and Conservation, Durrell Institute of Conservation and Ecology, University of Kent, Canterbury, United Kingdom; ^2^WildTeam Conservation, Allithwaite, United Kingdom; ^3^Wildwood Trust, Herne, Kent, United Kingdom; ^4^Re:wild, Austin, TX, United States

**Keywords:** work performance, burnout, work engagement, resilience, professional learning, conservation professionals, wellbeing, job characteristics

## Abstract

The purpose of this study was to explore how job characteristics relate to multiple dimensions of work performance in conservation professionals, with burnout and work engagement as mediating factors. A global sample of 561 conservation professionals across 98 countries completed an online survey. Using the Job Demands-Resources (JD-R) model and structural equation modelling, we found that participants with more frequent access to job resources (e.g., autonomy in work methods, social support at work, and availability of useful information), reported higher levels of work engagement. In turn, greater work engagement, characterised by vigour, dedication and absorption, was associated with increased task performance (e.g., working efficiently, managing time effectively) and contextual performance (e.g., taking initiative, creative problem-solving). Contrary to previous research in other sectors, job demands did not show a direct relationship to burnout in this conservation sample. However, higher burnout was linked to lower task performance. Moreover, burnout mediated the relationship between job resources and task performance: greater job resources were associated with lower levels of burnout, which in turn was associated with higher task performance. Our findings underscore the importance for both individual professionals and conservation organisations to enhance job resources and work engagement, given their positive relationships with multiple work performance indicators. These results may guide efforts to identify which perceived job characteristics are most likely to enhance performance, either directly or indirectly, through increased work engagement or reduced burnout. Furthermore, when organisations observe a decline across various performance indicators, this may signal a need to strengthen support for staff wellbeing and motivation. This study is the first to quantitatively examine relationships between job characteristics and multiple dimensions of work performance in a global sample of conservation professionals, highlighting the JD-R model’s relevance to conservation.

## 1 Introduction

The capacity of conservation professionals plays a vital role in safeguarding biodiversity and managing natural resources. Capacity refers not only to an individual’s knowledge and skills, but also to the combination and interaction of capacity on an individual, organisational and sectoral level that, taken together, influence work performance (1). Capacity development is the intentional process through which such capacity is created, strengthened and maintained over time (2, 3).

While most prior studies have focussed on the creation or strengthening of capacity (e.g., 4–12), comparatively little attention has been paid to understanding how it can be sustained in the long run. Recent work highlights how short-term and inflexible funding hinder conservation organisations’ ability to maintain institutional capacity over time (13). This vulnerability is further heightened by the sector’s exposure to economic and political instability. For example, the 2025 USAID funding cuts led to immediate and widespread job losses across hundreds of conservation projects globally (14). While organisations may attempt to adapt through alternative funding models, the professional and personal consequences for conservation staff are often profound. There is a significant overlap in countries with high biodiversity and those with limited financial and human capacity for conservation (15, 16). These stressors raise critical questions about the factors that support conservation professionals in remaining engaged and effective, particularly under pressure. Since organisational effectiveness ultimately relies on the performance of individual staff, understanding how their work environment supports or hinders that performance is vital (17–20).

Conservation professionals (hereafter also termed “conservationists”) work in diverse roles across varied ecosystems and institutional settings. Their responsibilities may include ecological research, law enforcement, education, or community engagement, often under conditions of limited resources and uncertainty. In many cases, their work requires balancing ecological priorities with complex socio-political realities (15), while navigating structural constraints such as understaffing, inadequate infrastructure, or lack of institutional support mechanisms (21–24). These challenges are further compounded by safety concerns and gender-based risks in certain contexts (2, 25, 26). This complex work environment underscores the need to examine how psychological processes influence conservation performance and well-being.

Although some studies have addressed psychological well-being in conservation (e.g., 24, 27), few have examined the predictors of sustained work performance. Most studies have focussed on isolated aspects of conservation capacity, such as skills, training, or motivation, without systematically examining the psychological mechanisms, such as work engagement and burnout, that may underpin work performance (28). The current study addresses this gap by applying the Job Demands-Resources (JD-R) model, a well-established framework from organisational psychology, to examine how job characteristics influence burnout, engagement, and work performance in conservation professionals worldwide (29).

### 1.1 The job demands–resources (JD-R model)

The Job Demands-Resources (JD-R) model is one of the most widely applied frameworks in occupational health psychology (30). This model has been tested across professional contexts and cultural settings and is used by government agencies to guide workplace health and safety policy (31). It offers a structured approach to understanding how job characteristics, i.e., the combination of job demands and job resources, shape employee engagement and stress-related outcomes. Job demands are defined as aspects of work requiring sustained cognitive, emotional, physical and/or behavioural effort (32), which can deplete energy and negatively affect mental wellbeing and performance (29, 33). In contrast, job resources are considered essential for generating the energy needed to respond effectively to job demands (32). They positively influence individuals’ energy levels and mental states (34), and contribute to enhanced performance (35). Job resources can be cognitive (e.g., access to relevant information and tools), social (e.g., collegial support during challenging situations), or physical (e.g., opportunities for rest and recovery during physically demanding tasks).

**Figure 1:**
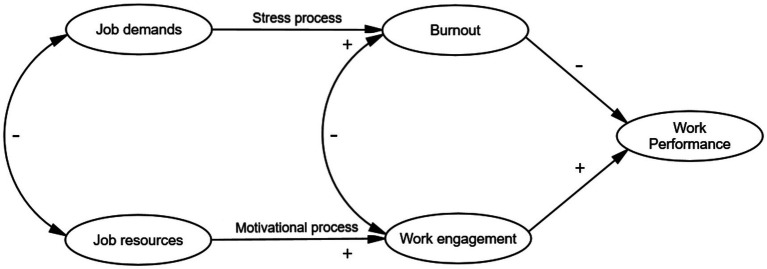
The Job Demands–Resources (JD–R) model, illustrating the relationships between job demands, job resources, burnout, engagement, and work performance. Based on Demerouti et al. (29) and Schaufeli and Bakker (36).

As outlined in Figure 1, the JD-R model proposes that when job demands are high and job resources are low, this combination may result in job stress and adverse health outcomes, such as exhaustion and burn-out, and is referred to as the stress process. Conversely, when job demands are low and job resources are high, the model predicts increased work engagement and favourable outcomes for health and work performance, known as the motivation process. The JD–R model has been applied in diverse sectors, including education, healthcare, and industry (37–39). In this study, we apply the framework to the conservation sector to inform a baseline for interventions that enhance staff wellbeing and performance, which are critical to realising long-term conservation impact.

### 1.2 Burnout

Burnout is a prolonged cognitive-emotional response to chronic work stressors, characterised by three dimensions: exhaustion, cynicism (depersonalisation) and reduced personal accomplishment (40). Exhaustion reflects the stress dimension of burnout and often leads individuals to emotionally and cognitively withdraw from their work, resulting in cynicism (40). Reduced personal accomplishment may follow from exhaustion and/or cynicism, or may emerge in parallel (40). Extensive psychology research has revealed several predictors of burnout, including high job demands, such as time pressure and workload (41) and emotional demands (42), as well as a lack of job resources, such as limited autonomy and insufficient social support (33).

Previous research has shown that job demands, as defined within the JD-R model, negatively affect conservation professionals’ stress levels, job satisfaction and conservation outcomes. These job demands included work overload and understaffing (15, 21, 22), and cognitive complexity, i.e., the extent to which a job is multifaceted and difficult to perform (15). Emotional demands may arise in roles that involve community-facing enforcement responsibilities, such as rangers tasked with implementing wildlife protection laws in the very communities to which they belong (21, 24). These situations often place professionals in morally complex positions that can heighten stress and strain relationships with local residents (22, 24).

Conservationists working in remote or high-risk environments also face physical strain and safety risks, including exposure to dangerous wildlife, armed groups, and gender-based violence, often in the absence of clear organisational reporting procedures and safeguarding policies (2, 23, 25–27, 43). These examples demonstrate how conservation-specific job demands align with the stress pathway outlined in the JD–R model and provide empirical grounding for our investigation into burnout. Based on this evidence, we hypothesise: (H1) job demands are positively related to burnout, and (H2) job resources are negatively related to burnout.

### 1.3 Work engagement

Work engagement refers to a positive, fulfilling state characterised by vigour, dedication, and absorption in one’s work (44). It is associated with beneficial outcomes for both individuals and organisations, including fewer health complaints and higher performance (35, 45, 46). Within the JD–R model, engagement is fostered by job resources, such as autonomy, social support, and access to relevant information, across sectors ranging from education and healthcare to telecommunications (30, 37, 39, 47). Organisational-level factors such as fairness, recognition, and efficient communication have also been shown to enhance engagement (37, 39). Additionally, in a study among public and private sector employees in Malaysia, Idris and Dollard (38) found that when management actively prioritised and communicated about occupational health and safety, which are characteristics of a strong psychosocial safety climate, this was associated with higher levels of engagement.

However, in the conservation sector, access to such resources is often constrained. Limited availability of essential tools, equipment, and information has been reported as a barrier to effective conservation work, particularly in the face of urgent biodiversity threats (15, 21–24). In a study involving 1,742 rangers across 293 sites in Africa, Asia, and Latin America, most respondents (68.1%) reported insufficient access to job resources such as proper equipment and basic amenities to ensure safety and fulfill their job requirements (23). Similarly, a survey of 114 conservation professionals in Vietnam found that 82.5% had experienced sexual harassment in the previous 2 years, and over half were unaware of any organisational procedure to report such incidents (25). These findings suggest that gaps in safety infrastructure and safeguarding mechanisms may undermine professionals’ engagement and well-being.

Social support, a key job resource in the JD–R model, has also been inconsistently available. Conservationists working in remote or socially isolated locations have reported limited peer support (23), while strained relationships with colleagues, marked by jealousy or anger, can further erode social cohesion (21). This lack of social and emotional support has been exacerbated where job responsibilities regularly prevented time with family (22, 24, 27). Conversely, support from supervisors and peers, including expressions of confidence, recognition, and connection to professional networks, has been shown to mitigate gender-related challenges among women conservation leaders (26, 48).

Perceptions of fairness in the workplace, termed organisational justice (49), has been identified as a key job resource that can help sustain work engagement. When professionals perceive fair access to promotions, recognition, and development opportunities, this may foster motivation, energy, and commitment to their work (27). In contrast, perceptions of injustice have been linked to dissatisfaction and disengagement. For example, Moreto (21) reported that the distribution of promotions was perceived as driven by favouritism, nepotism, and tribalism among rangers in Uganda, contributing to stress and dissatisfaction. Similar findings were observed in the Democratic Republic of Congo, where limited opportunities for promotion were associated with job dissatisfaction (22) and a reluctance among rangers across Africa, Asia and Latin America to recommend the profession to their children (23). Concerns about fairness and inclusion have also been raised by women conservation leaders in the US (26), and by professionals in biodiversity-rich, resource-constrained settings (27). Inequities in salary and advancement opportunities were reported to impede career development and job satisfaction (26), while experiences of recognition and appreciation were associated with enhanced energy and motivation (27).

Together, these findings support the motivational pathway outlined in the JD–R model. When conservation professionals experience job resources, such as social support, safety infrastructure, and organisational justice, they are more likely to remain engaged, which prompts our third hypothesis (H3): Job resources are positively related to work engagement. Furthermore, cross-sector studies have shown that work engagement declines with high physical demands, such as strenuous effort or exposure to hazards like noise, heat, and health risks (35), as well as with emotional demands (50). These findings support the hypothesis that (H4): job demands are negatively related to work engagement. Given that job demands are expected to increase burnout and job resources to enhance engagement, we propose a fifth hypothesis: (H5): job demands and job resources are negatively related.

### 1.4 Outcomes and work performance

Burnout and engagement have critical implications for the well-being and performance of conservation professionals. Evidence from studies across various sectors indicates that burnout is positively associated with absenteeism, lower productivity, and reduced organisational commitment, thereby increasing the likelihood of staff turnover (33, 34, 51). In contrast, engagement is linked to lower turnover intentions (52) and enhanced work performance (35, 46). Therefore, hypotheses 6, 7, and 8 are as follows: work engagement and burnout are negatively related (H6), burnout is negatively related to work performance (H7), and work engagement is positively related to work performance (H8).

Engaged employees tend to invest greater energy and focus in their work, reflected in multiple aspects of work performance, such as task performance, which refers to how competently individuals carry out the core or technical duties central to their job (53–55). Examples of task performance include goal setting, efficiency, and time management. In addition, engaged employees often demonstrate contextual performance, behaviours that support the psychological, social, and organisational environment, such as showing initiative, actively participating in team efforts, and taking on extra responsibilities (54, 56).

Work performance is therefore considered a multidimensional construct, encompassing task, contextual, and adaptive performance. Beyond the former two, adaptive performance refers to the ability to adjust effectively to changes in job roles and work environments (54, 57). This dimension is particularly relevant in conservation settings due to frequent exposure to uncertainty, adversity, and fast-changing environments (22, 27). Koopmans (54) developed a scale to quantify adaptive performance, including indicators of professional learning [e.g., updating one’s professional knowledge and skills; (58)], adapting positively to adversity [i.e., resilience; (59)], and creative and innovative problem-solving (60).

Despite the importance of performance in conservation, few empirical studies have measured it directly. Most have focused on related constructs such as stress, motivation, satisfaction, or barriers to success, often within specific subgroups such as rangers (21–24, 28, 43), or in studies addressing gender (26), country-specific contexts (25), or institutional constraints (15). With the exception of Ojha and Gairola (28), who quantitatively assessed the work performance of forest guards in India, none of these studies directly included self-reported measures of work performance. Building on Ojha and Gairola (28), the current study applied an updated measure of work performance suitable across sectors and job positions (61, 62) and different cultures (63). Earlier theories proposed a direct link between job satisfaction and performance (64), but more recent findings dispute this relationship (65, 66), and job satisfaction was therefore excluded from our study.

Burnout and engagement may also mediate the relationship between job characteristics and performance outcomes. For example, in a multi-sector study including health care, education, and the private sector, Bakker et al. (33) found that high job demands, especially workload, emotional strain, and work-home conflict, reduced individuals’ efficiency, as more energy and effort was required to maintain focus, ultimately impairing task performance. A lack of job resources, such as autonomy, social support, or professional development, has been shown to predict disengagement (cynicism), which in turn diminishes contextual performance (33). These mechanisms are consistent with the JD–R model and suggest that work engagement and burnout may serve as explanatory pathways. Research has supported this mediation framework across occupational settings (30), but it remains largely unexplored in conservation contexts.

Based on this evidence, we propose the following hypotheses:

(H9a): Burnout mediates the relationship between high job demands and work performance.

(H9b): Burnout mediates the relationship between low job resources and work performance.

(H10a): Work engagement mediates the relationship between high job resources and work performance.

(H10b): Work engagement mediates the relationship between low job demands and work performance.

## 2 Materials and methods

### 2.1 Participants and procedure

To test the study hypotheses, we conducted an online survey using convenience sampling (67) to collect data from conservation professionals. The survey was administered via Qualtrics (Qualtrics, Provo, USA) and distributed through the authors’ professional networks by emails and social media. Data were collected between 19 May 2019 and 20 January 2020. The survey included a participant information sheet emphasising the anonymity and confidentiality of the data. The questionnaire was designed in English and piloted with 20 individuals, including both native and non-native English speakers, and minor wording adjustments were made based on their feedback to ensure clarity and applicability to the conservation context.

A total of 561 valid responses were retained for analysis. Incomplete questionnaire submissions were excluded using listwise deletion, followed by the removal of 51 multivariate outliers identified via the Mahalanobis distance test [*p* ≤ 0.001; (68)]. This sample size exceeds power recommendations for structural equation modelling (SEM). According to MacCallum et al. (69), a sample of 200 is sufficient to achieve power > 0.90 for models with 100 degrees of freedom. Our final model included 1,174 degrees of freedom which, despite the inclusion of multiple latent variables and complex interrelations, indicates that our sample size provided adequate statistical power for our analysis.

### 2.2 Measures

The full questionnaire comprised 151 items, including demographics; only items relevant to the present study’s research questions are reported here (see Supplementary Table S1-1). In this study, we focused on a selection of job demands and job resources as predictors of work performance, while acknowledging that other factors may also influence the performance of conservation professionals. These predictors were selected based on constructs widely used in organisational psychology and were adapted for relevance to the conservation context. Job demands and resources were measured using established and validated scales [e.g. (70, 71)], including the Demand-Induced Strain Compensation (DISC) questionnaire (32, 72) and the Questionnaire on the Experience and Evaluation of Work [QEEW; (73)]. Work engagement and burnout were assessed using the Utrecht Work Engagement Scale [UWES-3; (74)], and a modified version of the Maslach Burnout Inventory-General Survey [MBI-GS; (75, 76)], respectively. In line with previous research (68, 77, 78), the inefficacy dimension of burnout in the MBI-GS was excluded, because it highly correlates with the vigour dimension of engagement in the UWES-3 scale and could be considered redundant. Work performance was measured using the International Work Performance scale [IWP; (54)], which captures task, contextual and adaptive performance. Some items were adapted or self-formulated based on prior qualitative research (27) to better reflect conservation-specific working conditions, since the physical demands and resources items in the DISC were originally developed for the nursing profession. Job demands, job resources and work performance items were scored on five-point Likert scales, ranging from “never” (1) to “always” (5) or “strongly disagree” (1) to “strongly agree” (5), with a “Not applicable” option provided where relevant (e.g., organisational support not available to independent contractors). Engagement and burnout items were rated on a seven-point scale from “never” (1) to “every day” (7), consistent with the original scales.

### 2.3 Analysis

We used structural equation modelling (SEM) to evaluate the JD-R model, which is an advanced multivariate technique that allows simultaneous testing of complex relationships between latent variables (79). SEM was selected over other statistical techniques because it accounts for measurement error, enables the testing of mediation effects, and models the latent structure of complex constructs, such as burnout and performance, that are represented by multiple observed indicators. This approach was consistent with our aim to examine the dual processes (stress and motivation) of the JD–R framework (29).

The 12 working conditions were classified into two latent factors, one representing job demands and the other job resources, and treated as exogenous (independent) variables in the model. In addition, the burnout, engagement, and the work performance variables were defined as endogenous (dependent) variables. The latent factors were allowed to correlate, following the rationale that working conditions also covary in reality, e.g., performance feedback with supervisor support (29). We followed a four-stage analytic process.

#### 2.3.1 Stage 1: preliminary analysis

We conducted multivariate analyses of variance (MANOVAs) using SPSS and Excel to examine demographic group differences. No significant effects were found for age, gender, country of residence and years of work experience.

#### 2.3.2 Stage 2: assumption testing

Assumptions underlying SEM were evaluated in a stepwise manner. Multivariate normality was tested using the squared Mahalanobis distance test (68). Based on a conservative probability estimate of *p* ≤ 0.001 (80), 51 outliers were identified and removed, resulting in a final sample size of 561 participants for analysis.

Multicollinearity was assessed using the Variance Inflation Factor (VIF), which detects whether two or more variables are highly correlated and may reflect the same underlying construct (68). All study variables showed acceptable levels of multicollinearity (Tolerance > 0.2; VIF < 5), suggesting that no pair of variables was highly correlated to the extent that they measured the same underlying construct (81).

Linearity and homoscedasticity were confirmed using scatterplots, which showed no systematic relationship between predicted values and the errors in the model (81). Finally, the assumption of homogeneity of variance was confirmed, by creating a variance chart in SPSS, to test the assumption that the variance of one variable was relatively similar to all levels of another variable. Observed variable variances ranged from 0.72 to 4.55, well below the threshold of 10, meaning no outliers were found (81).

#### 2.3.3 Stage 3: measurement model

To address common method variance, we followed recommendations by Conway and Lance (82). Three Confirmatory Factor Analyses (CFAs) were conducted on indicator variables derived from the multidimensional constructs: three job demands (9 items), six job resources (20 items), and three outcome variables (16 items). In each case, the assumed factor structure (see Supplementary Table S1-2) was compared to a one-factor solution. Where superior fit was found for the assumed factor model, fit was further improved using modification indices, which suggested allowing particular errors to correlate. To assess convergent and discriminant validity, we calculated average variance extracted (AVE) and composite reliability (CR) scores (83).

#### 2.3.4 Stage 4: structural model

**Figure 2:**
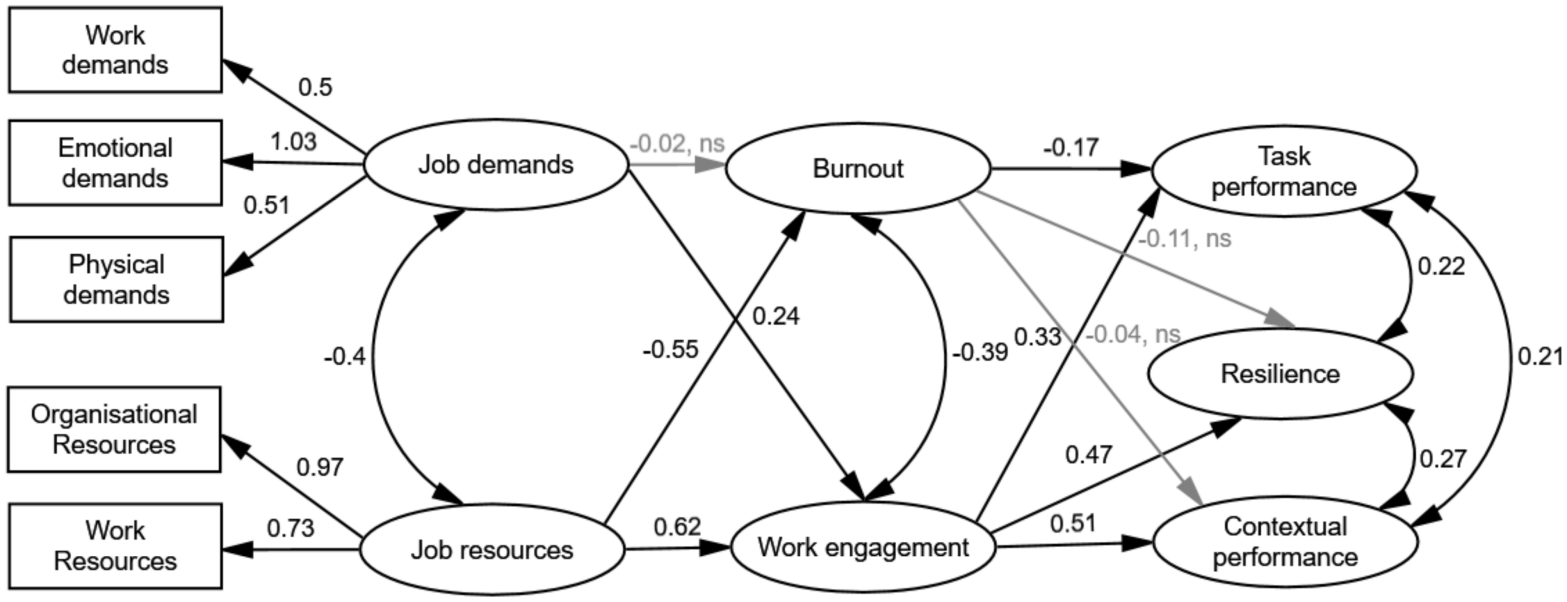
Structural equation modelling results of the final research model (M2). Lightgrey arrows represent non-significant (ns) pathways. Significant path coefficients (i.e., standardised regression weights) are represented along the black single arrows pathways and significant covariations are given next to the black double arrows (*p* < 0.05).

We then tested the research model (Figure 2) using SEM in AMOS 26.0 (84), employing maximum likelihood estimation. Model fit was evaluated using multiple indices: the chi-square (χ2) test statistic, Normed Fit Index (NFI), Tucker–Lewis index (TLI), Comparative Fit Index (CFI), and the Root Mean Square Error of Approximation (RMSEA). Thresholds for acceptable model fit were NFI, TLI and CFI > 0.90, and RMSEA ≤ 0.08 (68). RMSEA values > 0.10 were considered indicative of poor fit leading to model rejection (85). Statistical significance was set at *p* < 0.05.

## 3 Results

### 3.1 Characteristics of the participants

As noted in the analysis section, no significant demographic group differences were found based on MANOVAs (see Section 2.3.1). A total of 561 valid responses were retained for analysis after excluding incomplete questionnaire responses and after removing outliers using the Mahalanobis distance test (68). The majority of respondents were employed in conservation NGOs (56.33%), universities or research institutes (17.11%), and government agencies (13.55%). Participants included 257 men (45.81%) and 304 women (54.19%), with a mean age of 39 years (SD = 10.58). The sample represented professionals in 98 countries, with most holding a university degree (96.9%) or completing higher vocational training (2%) (Supplementary Table S1-3).

Internal consistency for all study variables exceeded the recommended threshold value of 0.70 (86), as presented in Supplementary Table S1-1. Table 1 provides descriptive statistics and correlations. An average score of all items per scale representing one variable are presented in percentages in this section and denoted with the average symbol (μ).

**Table 1:** Means, standard deviations, and correlations between all variables (*N* = 561).

	Variables	M	SD	1	2	3	4	5	6	7
1	Job Demands	4.38	0.67	—						
2	Job Resources	5.08	0.90	—0.31**	—					
3	Burnout	3.58	1.28	0.22**	—0.51**	—				
4	Engagement	5.26	1.04	0.14 *	0.35**	—0.46**	—			
5	Contextual performance	5.02	0.94	0.19**	0.18**	—0.23**	0.39**	—		
6	Task performance	5.16	0.87	—0.12 *	0.42**	—0.41**	0.36**	0.43*	—	
7	Resilience	4.98	0.94	0.02	0.36**	—0.35**	0.39**	0.45*	0.45**	—

M = Mean; SD = Standard Deviation; r = Pearson correlation coefficient. *p < 0.01, **p < 0.001.

### 3.2 Descriptive statistics

Participants reported high levels of work demands (M = 4.94, SD = 0.79), particularly for work overload (μ = 68.33%) and cognitive demands (μ = 75.28%), the latter including complexity and time pressure (Supplementary Table S1-1). In contrast, lower levels of social (M = 3.68, SD = 1.06) and physical demands (M = 3.61, SD = 1.35) were reported. Most participants reported never, rarely or occasionally encountering emotional demands (μ = 70.47%), such as unrealistic expectations, others’ anger or emotionally taxing problems. Similarly, on average 80.04% reported low levels of physical demands; however, a notable 30.48% indicated frequent exposure to physical safety risks from factors such as disease exposure, dangerous wildlife, or political instability (Supplementary Table S1-1).

Job resources were rated higher for work resources (M = 4.94, SD = 0.84) than organisational resources (M = 3.58, SD = 1.28). Work resources comprised cognitive, emotional and physical resources. In terms of cognitive resources, 20.68% of respondents reported never, rarely or occasionally having access to useful information (e.g., from computers, books, or co-workers) and 35.47% reported limited access to necessary tools (e.g., equipment, devices, software). Additionally, 37.47% lacked regular opportunities to alternate between complex and simple tasks, and 42.60% lacked mental breaks during cognitively demanding work. Regarding emotional resources, 60.25% indicated that co-workers were often or always willing to provide a listening ear during challenging situations. Nevertheless, 48.84% reported low levels of emotional support, and 49.02% felt unable to express emotions without fear of negative consequences. For physical resources, 35.29% reported limited ability to take breaks during physically strenuous work, 39.39% were rarely encouraged to discuss safety concerns, and 36.90% lacked opportunities to engage in safety-related activities ensuring the safest possible working conditions. Organisational resources encompassed communication, organisational justice, and recognition. While most respondents felt sufficiently informed about organisational developments (58.82%) and knew whom to approach for different problems (67.74%), fewer (47.42%) were clear on how decisions were made in their organisation. Regarding organisational justice, 43.49% of participants perceived the rewards they received for their work as fair, 53.83% believed that rules and procedures were applied fairly, and 70.94% felt they were treated fairly by their primary supervisor. A majority also felt respected at work (60.25%), valued for their skills (76.65%), and recognised and appreciated by their supervisor (75.58%).

Most respondents reported regular engagement at work (M = 5.26, SD = 1.04; μ = 78.55%). Nonetheless, 25–52% reported frequent (i.e., once a week to every day) symptoms of burnout (M = 3.58, SD = 1.28), i.e., mentally exhausted (52.41%), emotionally drained (40.82%), and cynical about whether their work contributes anything (25.31%).

Respondents reported high levels of task performance (M = 5.16, SD = 0.87); with 65–89% indicating they often or always complete tasks on time, set goals and priorities, work efficiently, and manage their time well. Nonetheless, around one-third reported lower performance on specific aspects, such as finishing work on time (34.22%), managing their time effectively (33.69%), and working efficiently (29.59%). Adaptive performance was similarly high (M = 5.12, SD = 0.83), with most respondents frequently updating their knowledge and skills (μ = 68.18%) and engaging in creative problem-solving or generating novel ideas (μ = 65.33%). However, a notable proportion reported these behaviours occurring less often, with about one third (μ = 31.82%) rarely updating their skills and infrequently using creativity in their work (μ = 34.67%). In terms of resilience, a component under adaptive performance, most respondents (μ = 70.32%) reported coping well with and recovering quickly from setbacks at work, while about one third indicated this was rarely the case (μ = 29.68%). Contextual performance received the highest scores (M = 5.17, SD = 0.78), with the majority of respondents (μ = 78.12%) often or always demonstrating initiative, actively participating, and taking on challenging or additional tasks.

### 3.3 Correlations

Table 1 presents the correlations between the higher order variables, i.e., job demands, job resources, burnout, engagement, contextual performance, task performance and resilience. This section reports the correlations between the lower order independent variables (i.e., work, emotional and psychical demands, and organisational and work resources) and the dependent outcome variables: burnout, engagement, contextual performance, task performance and resilience.

Job demands showed a significant positive correlation with burnout (r = 0.22, *p* < 0.001), specifically with work demands (r = 0.19, *p* < 0.001) and emotional demands (r = 0.32, *p* < 0.001). Physical demands were not significantly correlated with burnout (r = 0.14, ns). Job demands also demonstrated a significant positive relationship with engagement (r = 0.14, *p* < 0.01), particularly with work demands (r = 0.11, *p* < 0.01) and physical demands (r = 0.17, *p* < 0.001). No significant correlation was found between emotional demands and engagement (r = −0.06, ns). Contextual performance was significantly and positively associated with both work- (r = 0.14, *p* < 0.01) and physical demands (r = 0.14, *p* < 0.01), but not with emotional demands (r = −0.06, ns). Task performance was significantly and negatively associated with work demands (r = −0.11, *p* < 0.05) and emotional demands (r = −0.19, *p* < 0.001), while no significant relationship was observed with physical demands (r = −0.06, ns). Resilience showed a significant negative correlation with emotional demands (r = −0.12, *p* < 0.01), but was not significantly related to work (r = 0.05, ns) and physical demands (r = 0.01, ns).

Job resources showed a significant negative correlation with burnout (r = −0.51, *p* < 0.001), specifically with organisational resources (r = −0.51, *p* < 0.001) and work resources (r = −0.37, *p* < 0.001). Significant positive associations were found between job resources and engagement (r = 0.35, *p* < 0.001), including organisational resources (r = 0.34, *p* < 0.001) and work resources (r = 0.22, *p* < 0.001). Contextual performance had a significant positive correlation with organisational (r = 0.13, *p* < 0.001) and work resources (r = 0.15, *p* < 0.001). Similarly, task performance was significantly positively associated with both organisational (r = 0.35, *p* < 0.001) and work resources (r = 0.38, *p* < 0.001). Resilience also had a significant positive correlation with organisational (r = 0.29, *p* < 0.001) and work resources (r = 0.32, *p* < 0.001).

### 3.4 Model identification

Unidimensionality was ensured by setting the regression weight of the item with the largest loading value to 1. Confirmatory Factor Analysis (CFAs) were conducted next to assess the latent variable structure. Based on prior research in organisational psychology (87), it was expected that job demands would cluster into three dimensions: work demands, emotional demands, and physical demands. Job resources were expected to form three distinct factors as well: work resources, social resources, and organisational resources. Work performance outcomes were anticipated to cluster into task, contextual and adaptive performance dimensions (54).

To assess sampling adequacy for CFA, the Kaiser-Meyer-Olkin (KMO) measure was calculated. The KMO score of 0.89 indicated that the sample size (N = 561) was adequate for factor analysis, exceeding the recommended threshold of 0.5 (81). The fit of the three CFA models was improved significantly by allowing pairs of errors to correlate based on the Modification Indices. Specifically, three pairs of errors were allowed to correlate for job demands, fifteen for job resources, and thirteen for outcomes (Supplementary Table S1-2). These correlated errors represent common variance that is not explained by the latent construct and is most likely caused by overlapping items (87).

Following the CFAs, convergent and discriminant validity was assessed. Convergent validity, defined as the extent to which items load onto their intended latent variable, was tested using Average Variance Extracted (AVE) and Composite Reliability (CR) following Fornell and Larcker (83). Item loadings from AMOS were used to compute AVE and CR values in Excel. All variables met the criterion for composite reliability (CR > 0.70). However, only five of the eleven variables met the AVE threshold (>0.50), indicating that convergent validity was not established for six constructs: work demands, emotional demands, work resources, organisational resources, contextual performance, and adaptive performance. Next, discriminant validity was determined to ensure that each construct measured different characteristics. For each of the pairwise constructs, the squared correlations derived from AMOS were compared with the AVE scores, in which the AVE scores need to be greater than the squared correlations. Discriminant validity was established for all of the pairwise constructs, except for: (1) social resources with work resources, and (2) adaptive performance with contextual performance.

### 3.5 Model fitting

Model fitting modifications were made based on the outcomes of the convergent validity and discriminant validity assessments. The following items were deleted based on the low factor loading value (i.e., standardised regression weights) and corresponding AVE values: Work overload item 3 (0.42) from work demands; organisational justice item 1 (0.45) from organisational resources, and cognitive demand item 3 (0.30) and 4 (0.41) from work resources (88). Based on the results of the discriminant validity analysis, and in line with previous research (61, 63, 87), social resources were merged with work resources, and adaptive performance was integrated into contextual performance. An exception was made for two items related to resilience (AP1 and AP2), which were retained as a separate third dimension of work performance, as per Koopmans (54). These modifications resulted in an improved model (M2 modified), which demonstrated a better fit with the data. As shown in Table 2, the revised model achieved acceptable fit indices and was deemed sufficiently accurate in examining the causal effects between the constructs and can be applied to a much larger sample size.

**Table 2:** Test of research model.

	Model	*χ* ^2^	df	NFI	TLI	CFI	RMSEA
M1	Hypothesized model	3110.63	1,379	0.82	0.88	0.89	0.05
M2	Final model (modified)	2382.80	1,174	0.85	0.91	0.92	0.04

### 3.6 Model evaluation and modification

In this stage, the structural model was evaluated, the hypothesised model (M1) was tested and compared with a modified version (M2). Model testing results are presented in Table 2. The hypothesised model (M1) did not meet its criterion for all four goodness-of-fit indices, indicating poor model fit. To improve fit, the model was modified based on Modification Indices, specifically by allowing the pair of errors to correlate between burnout and engagement. This adjustment resulted in a revised model (M2) that demonstrated a better fit to the data compared to the original model (∆χ2 = 727.83, ∆df = 204). Although the Normed Fit Index (NFI) for M2 was slightly below the recommended threshold [NFI = 0.85; criterion ≥ 0.90; (68)], the overall model fit was considered acceptable. Specifically, the final model (M2) fits the data well based on the goodness-of-fit index (68) on the basis that (a) the RMSEA point estimate was below 0.05 (RMSEA = 0.04); (b) the upper bound of the 90% interval was 0.05, which is below the cut-off value of 0.06 proposed by Hu and Bentler (89), and the threshold 0.08 suggested by Browne and Cudeck (85); and (c) the associated probability value for the test of close fit was greater than 0.50 (*p* = 1.00). While the NFI value of 0.85 falls slightly below the commonly used threshold of 0.90, it is considered acceptable in light of the strong values obtained for other fit indices (e.g., CFI, RMSEA), which collectively support the adequacy of the model fit. Based on these indices, we concluded that the final model (M2) provided an adequate and well-fitting representation of the observed data.

### 3.7 Model testing

In the final stage, the structural relationships specified in the JD–R model were tested by evaluating the standardised path-coefficients and corresponding *p*-values. The majority of hypothesised relationships were significant and in the expected direction.

#### 3.7.1 Stress process

The initial path from job demands to burnout was positive and significant (γ = 0.22, *p* < 0.001). However, after including the hypothesised path from job resources to burnout, the association between job demands and burnout became non-significant (γ = −0.02, ns), and thus, hypothesis 1 was not supported. In contrast, job resources were negatively associated with burnout (γ = −0.55, *p* < 0.001), confirming hypothesis 2. Contrary to expectations, job demands showed a significant positive association with work engagement (γ = 0.24, *p* < 0.001), leading to the rejection of hypothesis 4, which posited a negative relationship. Regarding performance outcomes, burnout negatively predicted task performance (β = −0.17, *p* < 0.001), while its paths to contextual performance (β = −0.04) and resilience (β = −0.11) were non-significant. Given the merged constructs of adaptive and contextual performance (based on the discriminant validity results), hypothesis 7 was only partially supported: burnout negatively influenced task performance, but not contextual performance.

#### 3.7.2 Motivational process

All hypothesised paths within the motivational process were significant and positive. Job resources were positively related to engagement (γ = 0.62, *p* < 0.001), confirming Hypothesis 3. Engagement, in turn, positively predicted task performance (β = 0.33, *p* < 0.001), contextual performance (β = 0.51, *p* < 0.001), and resilience (β = 0.47, *p* < 0.001), thereby supporting Hypothesis 8.

The final JD–R model (M2), depicted in Figure 2, explained 46% of the variance in burnout and 58% in engagement, based on respective Average variance extracted (AVE) values. The explained variance in the outcome variables ranged from 41% for contextual performance to 54% for task performance. Significant negative covariations were found between job demands and job resources (β = −0.40, *p* < 0.001), and between burnout and work engagement (β = −0.39, *p* < 0.001), confirming hypotheses 5 and 6, respectively. Positive covariations were also found between the outcome variables: task and contextual performance (β = 0.21, *p* < 0.001), task performance and resilience (β = 0.22, *p* < 0.001), and contextual performance and resilience (β = 0.27, *p* < 0.001).

#### 3.7.3 Mediation analysis

Following Schaufeli (87), mediation effects were tested using Sobel’s (90) method. In the stress process, Hypothesis 9a, which proposed that burnout mediates the relationship between job demands and performance outcomes, was not supported, as no significant mediation effects were observed in the final model (M2). However, Hypothesis 9b was partially supported: burnout mediated the relationship between job resources and task performance (Sobel = 3.24, *p* < 0.01), but not contextual performance. This suggests that job resources reduce burnout, which in turn improves task performance.

Consistent with the motivation pathway, hypothesis 10a was supported: engagement mediated the relationship between job resources and task performance (Sobel = 4.50; *p* < 0.001), as well as contextual performance (Sobel = 7.20; *p* < 0.001). Hypothesis 10b was also supported: engagement mediated the relationship between job demands and task performance (Sobel = 3.34; *p* < 0.01) and contextual performance (Sobel = 3.82; *p* < 0.001). Additionally, engagement mediated the relationships between both job resources (Sobel = 6.64; *p* < 0.001) and job demands (Sobel = 3.73; *p* < 0.001) with resilience.

It should be noted that all data were self-reported, which may have introduced bias in the observed relationships; however, these patterns align with findings from prior multi-source studies (e.g., 33, 87).

## 4 Discussion

### 4.1 Burnout in conservationists

This study is the first to quantitatively examine the predictors of burnout and engagement, and their relationship to distinct work performance outcomes, in a broad sample of conservation professionals. We provide the first empirical model to describe influences on work performance across various conservation roles. Reported burnout levels (M = 3.58, SD = 1.28) were notably higher than those found among dentists in Finland [M = 1.70, SD = 1.18; (91)] and employees in other sectors in the Netherlands [M = 2.13, SD = 0.47; (33)], raising concern for the psychological wellbeing of conservationists.

Contrary to previous JD–R model applications (e.g. (92) for an overview), our results did not show a significant path from job demands to burnout. This may reflect the nature of the specific job demands assessed and the roles represented in the sample. For instance, due to the online format of the survey, professionals involved in intensive fieldwork may have been underrepresented, potentially leading to an underestimation of physically demanding conditions known to contribute to burnout.

The only significant consequence of burnout identified was its negative correlation with task performance, supporting prior findings (33). Burnout also partially mediated the link between job resources and task performance, offering partial support for H9b. In line with Bakker et al. (33), our findings suggest that higher job resources reduce burnout, which in turn enhances task performance. These findings highlight the importance of ensuring adequate resources to protect core operational effectiveness in conservation work.

### 4.2 Work engagement in conservationists

Significant pathways were found from job resources to engagement, and from engagement to both task and contextual performance, which is in line with empirical evidence across sectors (35). Engagement scores (M = 5.26, SD = 1.04) were higher than those reported among nurses in Canada [M = 3.90, SD = 0.89; (39)] and dentists in Finland [M = 4.46, SD = 1.07; (91)]. Engagement mediated the relationship between job resources and performance outcomes, aligning with the JD-R model’s motivational process (30) and confirming H10a.

Unexpectedly, a significant positive path was also found from job demands to engagement, with engagement mediating the relationship between job demands and both task and contextual performance (H10b). This suggests that, under certain conditions, job demands may be energising rather than depleting and these findings may be explained by the dual nature of job demands. Podsakoff et al. (93) proposed that job demands can be categorised into challenge and hindrance demands. Challenge demands, such as high workload, time pressure, responsibility, require substantial effort yet also hold potential for personal growth and achievement. These types of demands have been shown to correlate positively with both work engagement and burnout under certain conditions (78, 92). Hindrance demands, by contrast, such as role conflict, role ambiguity and role overload, impede goal achievement and tend to correlate positively with burnout and negatively with engagement (78, 92).

The job demands assessed in this study, including job complexity, level of attention required for tasks, time urgency and subjective workload, may, under certain conditions, be perceived as challenge stressors. This interpretation is supported by their positive correlations with both burnout and engagement and corresponds to empirical findings in other sectors. For instance, Hornung et al. (94) found job complexity to be positively associated with engagement among hospital staff, while its motivational potential has also been argued in earlier work on job design (35, 95). Although challenge demands can promote engagement, it is important to recognise that their positive effects may diminish or reverse if the individual becomes exhausted or lacks sufficient recovery time (78).

In conservation, demands such as urgent species interventions, high workloads during funding deadlines, or emotionally charged work with communities and ecosystems, may simultaneously foster engagement and increase the risk of burnout. This underscores the complexity of conservation work and the importance of job design and policy that maximises motivating aspects while mitigating risks.

### 4.3 Job demands predicting burnout and engagement

Among all demands measured, emotional demands showed the strongest positive correlation with burnout, yet a non-significant negative association with engagement. This pattern suggests that emotional demands, unlike complexity or workload, may act more as hindrance demands. Classification may, however, be context-specific. For example, Bakker and Sanz-Vergel (96) reported that nurses experienced emotional demands more as challenges, whereas de Jonge et al. (42) found them to predict emotional exhaustion in service sector employees (e.g., healthcare, recreation).

In our study, emotional demands were limited to interactions with people, not accounting for emotional tolls related to witnessing environmental destruction or species loss, factors known to affect conservationists’ energy, motivation and satisfaction (27). Forty to fifty percent of respondents reported feeling mentally exhausted and emotionally drained at least weekly. These findings indicate that emotional demands are a critical concern in conservation and should be more comprehensively captured in future research, including non-human triggers (e.g., habitat destruction, species extinction).

Physical demands were not correlated with burnout; however, we found a significant positive relationship between physical demands and engagement. Although initially surprising, this result can be interpreted in light of earlier findings. In a ranger-focused study, physical demands were appraised both as challenges and hindrances. Physically strenuous tasks were considered by some rangers to help maintain physical fitness, thereby enhancing engagement (i.e., functioning as a challenge demand), while physical safety concerns were seen as hindrances that obstructed job performance (21, 43). Physical demands will vary across job roles in conservation and so will subsequent consequences. Future studies should consider separating the physical demands assessed in this study into physically strenuous demands and physical safety risks, to better explore the conditions under which such demands act as hindrances or challenges across different conservation roles beyond law enforcement rangers.

### 4.4 Job resources predicting burnout and engagement

Job resources were the strongest predictor in the model, significantly enhancing engagement and reducing burnout. Under organisational resources, we examined communication, organisational justice (also “fairness”), and recognition and appreciation. Previous research has highlighted the significant role of perceived fairness in organisational policies and administration, such as funding allocation and career development opportunities, in shaping the psychological wellbeing of conservation professionals (21, 26, 27). This insight is echoed in other professions, where a lack of perceived fairness has been linked to burnout and health risks among university and hospital staff (97, 98), while fairness was positively associated with engagement among nurses (39). Organisational communication includes transparency about important developments, decision-making procedures, and information on where to find support when problems arise. Feeling recognised, respected, and appreciated for one’s work is also essential. Together, these aspects are closely linked to the notion of fairness and can help create empowering work conditions that foster staff engagement (26, 39). These organisational resources are therefore often grouped under the umbrella of fairness within the JD–R model (87). As organisations increasingly explore flexible working models, allowing staff to choose where and when to work, effective and efficient communication becomes even more important in maintaining high levels of work engagement (37).

Work resources were also significantly correlated with both burnout (negatively) and engagement (positively), confirming their dual role in supporting wellbeing and performance. In this study, work resources encompassed cognitive, social, and physical aspects that support day-to-day conservation work. The vast majority of respondents in this study reported not always having access to the information, tools and equipment needed to perform their jobs effectively. Many also lacked opportunities to take mental breaks during periods of high concentration, an essential resource for maintaining cognitive functioning and preventing overload. These findings echo those from ranger-focused studies, which emphasise the critical need for access to appropriate tools and “perishable” equipment, such as boots, rain jackets, mosquito nets, tents, and, where applicable, weapons and ammunition. These studies also showed that basic amenities like sufficient clean drinking water and suitable foods were not always present for safeguarding wellbeing and enabling effective field operations (21–23). While prior research has largely focused on law enforcement roles, our findings indicate that similar resource gaps affect a broader range of conservation professionals, including those in desk-based or hybrid roles, potentially undermining their wellbeing and effectiveness in less visible ways.

Social support also played a key role in our findings. Although a majority of respondents indicated that colleagues were often willing to listen during challenging situations, nearly half reported limited access to emotional support or felt unable to express their emotions freely at work. These results are consistent with previous studies that have shown social support to be a crucial buffer in conservation workplaces, especially in settings characterised by interpersonal conflict or isolation, e.g., limited time with friends and family (21–24, 26, 27). Our findings reinforce the need for organisations to foster emotional safety at work, not only by enabling supportive peer relationships, but also by cultivating a culture in which staff feel valued, heard, and cared for.

Staff who lack key resources experience negative consequences, such as heightened burnout, poorer task performance, and reduced emotional and operational safety, that mirror findings from previous studies in conservation. For example, inadequate support was linked to serious risks including harassment, retaliation by local communities, and reduced job effectiveness due to poor decision-making or enforcement in situations where employees were not supported, recognised and respected by superiors (21, 22, 25, 26, 99). Our findings reflect these same patterns at a broader scale, as respondents who reported lacking cognitive, social, and physical work resources also reported higher burnout and lower task performance. This suggests that the risks associated with insufficient support extend beyond field-based roles and may be embedded more widely across the sector.

### 4.5 Resilience

Despite the significant negative correlation from burnout to resilience (r = −0.35, *p* < 0.001), we found no evidence that burnout reduced resilience, since the path-coefficient from burnout to resilience in our final model (M2) was nonsignificant. In contrast, job resources and work engagement were both significantly and positively related to resilience and so was the path-coefficient from work engagement to resilience (β = 0.41). This supports findings from Kašpárková et al. (65), who showed that resilient employees in helping professions, including health care, education, social work, tended to be more engaged, more satisfied and demonstrated higher work performance than their less resilient peers. They also found that work engagement partially mediated the relationship between resilience and work performance. Our study showed significant and positive covariations between resilience and both task and contextual performance, though cause-effect directionality was not confirmed. These findings suggest that resilience may play an important role in enabling conservation professionals to maintain high performance under pressure, particularly when supported by sufficient job resources. Future research could benefit from longitudinal or experimental designs to better understand the direction and mechanisms of this relationship, especially within high-stakes conservation settings.

Descriptive results further indicated that approximately 30% of respondents struggled to cope effectively with difficult situations or to recover promptly from setbacks. This aligns with earlier findings from the conservation field, which highlighted a growing need for individual and systemic resilience strategies to help professionals thrive in the face of complex and emotionally taxing work conditions (2, 27). The high-stakes, value-driven nature of conservation work means that exposure to emotionally demanding or ethically conflicting situations is often unavoidable, further underscoring the need for targeted support.

In line with the motivational process of the JD–R model, our results indicate that resilience can be strengthened by increasing access to job resources that also foster engagement, such as feedback, autonomy, access to tools, and social support. This concurs with research among healthcare professionals, where resilience was found to be associated with healthier coping behaviours and improved workplace wellbeing (100). These findings carry important policy implications. We recommend that conservation organisations prioritise resilience-building not only at the individual level but also through structural strategies, such as embedding mentoring systems, allowing time for recovery and reflection, and designing workflows that support long-term wellbeing.

Evidence-based interventions, such as short-term workplace coaching, have also shown promise in enhancing resilience and improving workplace wellbeing (101). Providing conservation professionals with access to such resources, whether through organisational offerings or external partnerships, could offer a cost-effective means of maintaining performance and protecting wellbeing across diverse roles and regions.

## 5 Conclusion

This study provides new empirical insights into the predictors of work performance, burnout, and engagement among conservation professionals. Among our global sample of 561 professionals, job resources were the strongest predictors of work performance, operating through both the stress process (affecting task performance) and the motivational process (affecting both task and contextual performance). Job resources also played a central role in supporting resilience, underscoring their broader importance for sustaining wellbeing in demanding conservation contexts.

The stress and motivation processes were clearly present in our sample: burnout was negatively associated with task performance, while engagement was positively associated with both performance outcomes and resilience. These are distinct processes that require tailored strategies (36). One cannot prevent burnout merely by providing more job resources if individuals are already exposed to sustained cognitive, emotional, or physical overload. Rather, burnout prevention requires reducing stressors, while engagement must be actively fostered through different mechanisms. Conservation organisations must therefore adopt a dual approach: minimising job demands that contribute to burnout (e.g., interpersonal conflict, lack of recovery time) and simultaneously enhancing the job resources that promote engagement (e.g., autonomy, recognition, and transparent communication). We recommend team-level interventions in particular, given evidence of crossover effects, where both burnout and engagement can spread between colleagues (102).

While job demands are often associated with strain, not all demands have the same impact. A useful distinction is that between challenge demands, which can promote learning and engagement, and hindrance demands, which tend to obstruct performance and lead to exhaustion (92, 93). Our findings illustrate this dynamic: for example, cognitive demands were linked to both burnout and engagement, suggesting that some demands may energise professionals under the right conditions, but contribute to strain when certain job resources (e.g., access to relevant information) are lacking. Whether a demand functions as a challenge or a hindrance varies across occupations and individuals (96). Conservation organisations and their employees may benefit from identifying the challenge and hindrance demands relevant in their work and work environment. Organisations can reduce the stress process by eliminating hindrance demands, e.g., interpersonal conflicts, whilst ensuring that challenge demands contribute to engagement rather than employee exhaustion by monitoring employees’ stress levels (78). These contextual variations are especially relevant in conservation, given the diversity of roles and settings across the sector.

To strengthen the motivational process, conservation organisations could consider job redesign strategies that improve access to tools and information, increase autonomy, and expand opportunities for participation in decision-making. Just as vital is organisational support and recognition, including transparent reward systems and fair distribution of professional growth opportunities. These actions are essential for enabling individuals and teams to thrive, especially in the face of complex and resource-limited working environments.

We strongly recommended that conservation organisations, funders, and sector leaders make workforce wellbeing a core pillar of conservation strategy and policy. This includes routinely assessing working conditions, investing in staff support systems, embedding resilience-building practices into day-to-day operations, and ensuring equitable treatment and support across roles and regions. The 2025 USAID funding cuts, which led to widespread job losses across conservation projects, highlight the vulnerability of conservation professionals to systemic shocks, and the urgent need to buffer their wellbeing and performance through more resilient organisational models (14). By prioritising job design, support structures, and engagement strategies, organisations can not only strengthen individual and team performance, they also build institutional capacity that is better able to withstand political and financial uncertainty. In doing so, the sector can move toward a more effective, equitable, and sustainable conservation future.

### Applicability of JD-R model and study limitations

Despite the ambiguity on whether a specific job characteristic represents a challenge or hindrance demand in the JD-R model (31), we found this framework to be a valuable tool for understanding burnout and motivation processes in the conservation workforce. The JD-R model makes these psychological processes accessible to organisational intervention (33). For example, when managers are able to reduce hindrance demands, such as by assigning a manageable workload, employees’ task performance may improve via increased engagement.

However, researchers should approach the model with contextual sensitivity. Gaining sufficient understanding of how specific job characteristics function in conservation work is necessary to ensure appropriate application of the model’s assumptions. We also recommend future research to include other organisational resources shown to influence conservationists’ motivational and stress responses, such as relationships with line managers and opportunities for professional growth and development (27).

The main limitations of this study concern its cross-sectional design and the exclusive use of self-reported measures. As a result, we could not test for causality and longitudinal studies are needed to further validate the findings. For example, a longitudinal study on the stress process of staff at an employment agency showed that work pressure, work-home interference and exhaustion each predicted each other over time and therefore none of these could be considered only a predictor or only an outcome (41). Exploring reciprocal relationships between job characteristics and outcomes would therefore be a valuable next step. Additionally, the use of self-reported measures may have introduced a positive bias in the associations among the study concepts (103). Nevertheless, previous studies that relied on supervisor-or peer-rated work performance found similar correlations between job characteristics and outcomes, suggesting that our results are consistent with other-rated performance assessments (33, 87).

Finally, although no significant differences were found in burnout, engagement, or work performance across demographic variables, such as age, gender, country of residence, or years of work experience, the fact that our survey was available exclusively in English and online limits the generalisability of the sample. The influence of demographics or regional differences cannot be fully excluded. Whilst this is a notable limitation, our findings still offer valuable insights into the experience of conservation professionals across a wide geographic and institutional spectrum.

## Data Availability

The datasets presented in this article are not readily available because of potential identifiable data and the need to protect the privacy and anonymity of the participants. Requests to access the datasets should be directed to Thirza Loffeld, thirzaloffeld@gmail.com.
