# Supplementary material for: From burnout to engagement: enhancing the wellbeing and performance of conservationists
Source: Front Vet Sci. 2025 Sep 19;12:1567931. doi: 10.3389/fvets.2025.1567931 (PMC12492983; doi:10.3389/fvets.2025.1567931)
Supplement: Supplementary file 1 [file Table_1.docx]

## **Loffeld et al. 2025. Supplementary information**

**Table S1-1: questionnaire items.**

| **Construct** | | **No** | **α** | **Example item** | **Source** |
| --- | --- | --- | --- | --- | --- |
| **Job demands** | | **12** |  |  |  |
|  | *Work demands* |  |  | *Cognitive demands* | |
| 1 | Cognitive demands  and work overload | 6 | 0.74 | I have to solve work-related problems within a limited time frame | DISC-S 2.1 (de Jonge et al. 2009) |
|  | *Emotional demands* |  |  | *Emotional demands* | |
| 2 | Emotional demands | 3 | 0.72 | I have to deal with people (e.g. beneficiaries, colleagues or supervisors) whose problems touch me emotionally | DISC-S 2.1 (de Jonge et al. 2009) |
|  | *Physical demands* |  |  | *Physical demands, incl. safety culture* |  |
| 3 | Physical demands | 3 | 0.82 | In my work I am exposed to physical safety issues due to external factors (e.g. disease pressure, dangerous wildlife, political instability) | 1 item from DISC-S 2.1 (de Jonge et al. 2009) and 2 items self-developed based on Loffeld et al. (2022) |
| **Job resources** | | **20** |  |  |  |
|  | *Work resources* |  |  |  |  |
| 1 | Cognitive Resources | 5 | 0.71 | I have the opportunity to determine my own work method. | DISC-S 2.1 (de Jonge et al. 2009) and 1 item from Schaufeli (2015) |
| 2 | Physical Resources | 3 | 0.78 | In my work, I have the opportunity to actively engage in meaningful safety related activities | 1 item DISC-S 2.1 (de Jonge et al. 2009), 2 items self-developed based on Loffeld et al. (2022) |
|  | *Social resources* |  |  |  |  |
| 1 | Emotional Resources | 3 | 0.88 | I get emotional support from others (e.g. colleagues, supervisors or beneficiaries) when a challenging situation at work occurs. | DISC-S 2.1 (de Jonge et al. 2009) |
|  | *Organisational resources* |  |  |  |  |
| 1 | Communication | 3 | 0.87 | I am sufficiently informed about important developments within my organisation | QEEW (Veldhoven et al. 2002) |
| 2 | Organisational justice | 3 | 0.73 | In my opinion, the rules and procedures at work are applied in a fair manner | Jordan and Turner (2008) |
| *3* | Recognition and Appreciation | 3 | 0.80 | Working with members of this team, my unique skills and talents are valued | 1 item from DISC-S 2.1 (de Jonge et al. 2009), 1 item from Edmondson (1999), 1 item modified from QEEW (Veldhoven et al. 2002) |
| **Employee wellbeing** | | **9** |  |  |  |
|  | *Burnout* |  |  |  |  |
| 1 | Exhaustion | 3 | 0.91 | I feel mentally exhausted because of my work | MBI-GS (Schaufeli et al., 1996) |
| 2 | Cynicism | 3 | 0.90 | I have become more cynical about whether my work contributes anything | MBI-GS (Schaufeli et al., 1996) |
|  | *Work engagement* |  |  |  |  |
| 1 | Vigour  Dedication Absorption | 3 | 0.79 | I am bursting with energy and vigour at work | UWES-3 (Schaufeli et al. 2017) |
| **Outcomes** | | **16** |  |  |  |
|  | *Work performance* |  |  |  |  |
| 1 | In-task performance | 5 | 0.85 | I was able to plan my work so that I finished it on time | IWP (Koopmans, 2014) |
| 2 | Contextual performance | 4 | 0.77 | I took on extra responsibilities | IWP (Koopmans, 2014) |
| 3 | Adaptive performance | 7 | 0.85 | I worked on keeping my job-related knowledge up-to-date | IWP (Koopmans, 2014) |

**Table S1-2: CFA goodness-of-fit outcomes** for the independent variables (i.e. job demands and job resources) and dependent variables (i.e. work performance) evaluated using the chi-square (χ2) test statistic, the Normed Fit Index (NFI), the Tucker–Lewis index (TLI), the comparative fit index (CFI), and the root mean square error of approximation (RMSEA).

| Model | χ2 | df | NFI^1^ | TLI^1^ | CFI^1^ | RMSEA^1^ |
| --- | --- | --- | --- | --- | --- | --- |
|  |  |  |  |  |  |  |
| *Job demands* |  |  |  |  |  |  |
| 1-factor | 891.28 | 54 | 0.53 | 0.44 | 0.54 | 0.17 |
| 3-factor | 137.52 | 51 | 0.93 | 0.94 | 0.95 | 0.06 |
| 3-factor (modified) | 95.31 | 48 | 0.95 | 0.97 | 0.97 | 0.04 |
|  |  |  |  |  |  |  |
| *Job resources* |  |  |  |  |  |  |
| 1-factor | 2106.61 | 170 | 0.61 | 0.59 | 0.63 | 0.14 |
| 2-factor | 1423.87 | 169 | 0.74 | 0.73 | 0.76 | 0.12 |
| 2-factor (modified) | 287.59 | 149 | 0.95 | 0.97 | 0.97 | 0.04 |
|  |  |  |  |  |  |  |
| *Outcomes* |  |  |  |  |  |  |
| 1-factor | 2055.08 | 104 | 0.54 | 0.48 | 0.55 | 0.18 |
| 3-factor | 1048.19 | 101 | 0.77 | 0.74 | 0.78 | 0.12 |
| 3-factor  (modified) | 249.41 | 92 | 0.95 | 0.95 | 0.96 | 0.05 |
| Note: n = 561 |  |  |  |  |  |  |

^1^Values larger than 0.90 for NFI, TLI and CFI and 0.08 or lower for RMSEA indicate acceptable model fit (Byrne, 2016). For RMSEA, values greater than 0.10 should lead to model rejection (Browne & Cudeck, 1993).

**Table S1-3: Percentage of respondents (n = 561) per country of residence. Half of the respondents were based in biodiversity-rich countries with limited access to resources.**

|  | **Country of residence** | **Percentage of respondents (%)** |
| --- | --- | --- |
| 1 | Albania | 0.2 |
| 2 | Argentina | 0.9 |
| 3 | Australia | 2.4 |
| 4 | Austria | 0.3 |
| 5 | Bahamas | 0.2 |
| 6 | Bangladesh | 0.5 |
| 7 | Belarus | 0.3 |
| 8 | Belgium | 0.2 |
| 9 | Belize | 0.2 |
| 10 | Benin | 0.5 |
| 11 | Bolivia | 0.9 |
| 12 | Bosnia and Herzegovina | 0.2 |
| 13 | Brazil | 2.7 |
| 14 | Burkina Faso | 0.2 |
| 15 | Cambodia | 0.5 |
| 16 | Cameroon | 0.9 |
| 17 | Canada | 2.9 |
| 18 | Central African Republic | 0.2 |
| 19 | Chile | 0.2 |
| 20 | China | 0.3 |
| 21 | Colombia | 1.2 |
| 22 | Congo, Republic of the... | 0.2 |
| 23 | Costa Rica | 0.3 |
| 24 | Côte d'Ivoire | 0.3 |
| 25 | Croatia | 2.4 |
| 26 | Czech Republic | 0.2 |
| 27 | Democratic Republic of the Congo | 0.3 |
| 28 | Denmark | 0.2 |
| 29 | Dominican Republic | 0.2 |
| 30 | Ecuador | 0.3 |
| 31 | El Salvador | 0.5 |
| 32 | Ethiopia | 0.3 |
| 33 | Fiji | 0.2 |
| 34 | Finland | 0.2 |
| 35 | France | 1.2 |
| 36 | Germany | 0.7 |
| 37 | Ghana | 0.5 |
| 38 | Greece | 0.5 |
| 39 | Guatemala | 0.7 |
| 40 | Guinea | 0.2 |
| 41 | Guyana | 0.2 |
| 42 | Honduras | 0.2 |
| 43 | Hungary | 0.2 |
| 44 | India | 3.2 |
| 45 | Indonesia | 3.9 |
| 46 | Ireland | 0.2 |
| 47 | Italy | 0.3 |
| 48 | Jordan | 0.5 |
| 49 | Kenya | 2.0 |
| 50 | Lao People's Democratic Republic | 0.3 |
| 51 | Liberia | 0.5 |
| 52 | Luxembourg | 0.2 |
| 53 | Madagascar | 0.9 |
| 54 | Malawi | 1.0 |
| 55 | Malaysia | 3.9 |
| 56 | Malta | 0.2 |
| 57 | Mauritius | 0.9 |
| 58 | Mexico | 0.9 |
| 59 | Mongolia | 0.2 |
| 60 | Morocco | 0.3 |
| 61 | Mozambique | 0.2 |
| 62 | Myanmar | 0.3 |
| 63 | Namibia | 0.5 |
| 64 | Nepal | 0.7 |
| 65 | Netherlands | 0.7 |
| 66 | New Zealand | 0.5 |
| 67 | Nigeria | 1.4 |
| 68 | Norway | 0.3 |
| 69 | Pakistan | 0.3 |
| 70 | Peru | 0.5 |
| 71 | Philippines | 0.2 |
| 72 | Portugal | 2.7 |
| 73 | Russian Federation | 0.2 |
| 74 | Rwanda | 1.2 |
| 75 | Saint Kitts and Nevis | 0.2 |
| 76 | Saint Lucia | 0.2 |
| 77 | Samoa | 0.5 |
| 78 | Seychelles | 0.7 |
| 79 | Singapore | 1.7 |
| 80 | Slovakia | 0.2 |
| 81 | South Africa | 4.1 |
| 82 | Spain | 0.3 |
| 83 | Sri Lanka | 0.3 |
| 84 | Suriname | 0.2 |
| 85 | Sweden | 0.2 |
| 86 | Thailand | 1.4 |
| 87 | Uganda | 1.0 |
| 88 | Ukraine | 0.2 |
| 89 | United Arab Emirates | 0.2 |
| 90 | United Kingdom of Great Britain and Northern  Ireland | 18.5 |
| 91 | United Republic of Tanzania | 0.9 |
| 92 | United States of America | 12.2 |
| 93 | Venezuela, Bolivarian Republic of... | 0.3 |
| 94 | Viet Nam | 0.7 |
| 95 | Zambia | 0.7 |
| 96 | Zimbabwe | 0.2 |
| 97 | Falkland Islands | 0.2 |
| 98 | Saint Helena, Ascension and Tristan da Cunha | 0.5 |
|  | Total | 100 |
